# The Patriarch’s Letter

**Published:** 1857-09

**Authors:** Grant Thorburn

**Affiliations:** New Haven


### THE PATRIARCH'S LETTER.

My manner of life, from my youth up, I think, has been dictated by the laws of Nature. From my earliest recollection I never could swallow any food or drink hotter or colder than the blood, without a painful sensation, nor drink spirits, as it burnt my mouth and throat. Some seventy years ago, by the help of a powerful microscope, I saw scores of living things swimming in a tumbler of water. Thinks I to myself, if we must swallow whole fish, we had better boil them first. I think every thing that goes into the stomach ought to pass through the fire.

I love all kinds of fruit, after a fiery trial in a pie dish, pudding-bag, or dumpling; but I can't recollect that I have eaten a raw apple or peach in seven years. The good fruit and vegetables I delighted to honor at my table fifty years ago, are as much my favorites now as they were then. The reason is obvious. In that long period I never ate enough. My young unsatisfied appetite continued to say, "Give, give!" In 1802 I kept a retail grocery in New York. Often in the middle of my dinner I was called to wait on customers; being detained fifteen or twenty minutes, on going back to the table my appetite was gone; I returned to the store and ate no more until tea time. Observe, had I not been called off, I would have eaten the whole plateful, before rising from the table. This thing often occurring, I noticed that I always felt more comfortable through the afternoon, and never wished for my tea until the usual hour. So I put myself on short allowance, on which I continue to live, move, and have my being, at the present day. When I see people at table heap their plates with fish, flesh, and fowl, potatoes, cabbage, cucumber, and sour krout, apple, mince, and custard pies, says I to myself, "They are digging their graves with their teeth." GRANT THORBURN, SR.,

"NEW HAVEN, 18*th July*, 1857. *Aged* 84 *years*, 5 *months*.

